# Rapid systemic surge of IL-33 after severe human trauma: a prospective observational study

**DOI:** 10.1186/s10020-021-00288-1

**Published:** 2021-03-26

**Authors:** Olav Sundnes, William Ottestad, Camilla Schjalm, Peter Lundbäck, Lars la Cour Poulsen, Tom Eirik Mollnes, Guttorm Haraldsen, Torsten Eken

**Affiliations:** 1 K.G Jebsen Inflammation Research Centre, Institute of Clinical Medicine, Faculty of Medicine, University of Oslo, Oslo, Norway; 2 Department of Pathology, Oslo University Hospital, Rikshospitalet, N-0027 Oslo, Norway; 3 Department of Dermatology, Oslo University Hospital, Oslo, Norway; 4 Department of Anaesthesiology, Division of Emergencies and Critical Care, Oslo University Hospital Ullevål, Oslo, Norway; 5 Division of Critical Care, Institute of Clinical Medicine, Faculty of Medicine, University of Oslo, Oslo, Norway; 6 Department of Immunology, Oslo University Hospital, Oslo, Norway; 7 Reserach Laboratory, Nordland Hospital, Bodø, and K.G.Jebsen TREC, University of Tromsø, Tromsø, Norway; 8 Centre of Molecular Inflammation Research, Norwegian University of Science and Technology, Trondheim, Norway

**Keywords:** Alarmins, Interleukin-33, Biomarkers, Immunity, Innate, Wounds and Injuries, Kinetics, Humans, Critical Care, Prospective Studies

## Abstract

**Background:**

Alarmins are considered proximal mediators of the immune response after tissue injury. Understanding their biology could pave the way for development of new therapeutic targets and biomarkers in human disease, including multiple trauma. In this study we explored high-resolution concentration kinetics of the alarmin interleukin-33 (IL-33) early after human trauma.

**Methods:**

Plasma samples were serially collected from 136 trauma patients immediately after hospital admission, 2, 4, 6, and 8 h thereafter, and every morning in the ICU. Levels of IL-33 and its decoy receptor sST2 were measured by immunoassays.

**Results:**

We observed a rapid and transient surge of IL-33 in a subset of critically injured patients. These patients had more widespread tissue injuries and a greater degree of early coagulopathy. IL-33 half-life (t_1/2_) was 1.4 h (95% CI 1.2–1.6). sST2 displayed a distinctly different pattern with low initial levels but massive increase at later time points.

**Conclusions:**

We describe for the first time early high-resolution IL-33 concentration kinetics in individual patients after trauma and correlate systemic IL-33 release to clinical data. These findings provide insight into a potentially important axis of danger signaling in humans.

**Supplementary Information:**

The online version contains supplementary material available at 10.1186/s10020-021-00288-1.

## Background

It is well established that the immune system not only discriminates between self and non-self but also responds to insults that affect tissue homeostasis. This concept has been clearly demonstrated in experimental models of sterile tissue damage, shock and reperfusion (Bianchi 2007; Levy et al. 2007; Matzinger 2002). In patients with severe trauma and shock the innate immune response can lead to unbridled systemic inflammation with subsequent multiple-organ failure (MOF) and adverse outcomes (Stoecklein et al. 2012). The immune response after such tissue damage is thought to be initiated by alarmins, a diverse group of constitutively expressed molecules that are released upon cellular damage (Hirsiger et al. 2012; Hwang et al. 2011). As they are likely the most proximal mediators of the immune response to injury, there is great interest in understanding their biology and their potential as future biomarkers or therapeutic targets in a variety of conditions, including the inflammatory sequelae following major trauma.

Interleukin-33 (IL-33) is a novel IL-1 family member known to have pleiotropic effects in both innate and adaptive immunity (Martin and Martin 2016; Molofsky et al. 2015). IL-33 possibly exerts dual functions: one as a nuclear factor and another as an extracellular cytokine signaling through its receptor ST2 (Carriere et al. 2007; Haraldsen et al. 2009). It thus resembles other dual-function alarmins such as HMGB1 and IL-1α (Haraldsen et al. 2009). Extensive research to understand IL-33 biology has largely been performed in murine models but its regulation and release to the extracellular environment in human physiology and pathophysiology remains unclear (Martin and Martin 2016). While cellular necrosis appears to be the main mechanism of release, active secretion has also been described (Kakkar et al. 2012; Kouzaki et al. 2011).

We have recently published data on the early concentration kinetics of the prototype alarmin HMGB1 in human trauma patients (Ottestad et al. 2019). Unlike HMGB1, which is nearly ubiquitously expressed (Müller et al. 2004), human IL-33 is restricted to constitutive expression in endothelial cells along the vascular tree, epithelial cells at certain barrier surfaces, and varying constitutive expression in other stromal cell types (Küchler et al. 2008; Pichery et al. 2012). Whether the strong constitutive vascular expression pattern reflects an intracellular function in quiescent endothelial cells is not yet clear, but this location nevertheless indicates that IL-33 is ideally positioned for rapid systemic release upon endothelial injury. Current knowledge of IL-33 biology is largely derived from experimental models in mice. The translational value is limited since mice have a markedly different expression pattern compared to humans (Pichery et al. 2012; Sundnes et al. 2015) and importantly, in the context of tissue injury, lack widespread constitutive expression of IL-33 in the vasculature (Pichery et al. 2012).

The biological activity of IL-33 is tightly regulated and, as described for other IL-1 family members (Granowitz et al. 1991), one level of activity control is the synthesis of a soluble decoy receptor (sST2) (Pascual-Figal and Januzzi 2015). sST2 is abundantly expressed in blood in healthy individuals, with increased levels detected in a variety of conditions. Previous studies have shown that levels of circulating sST2 are markedly increased in trauma patients (Brunner et al. 2004; Du et al. 2018; Xu et al. 2017). IL-33, on the other hand, appears to be present in very low or undetectable levels in plasma or serum. A few recent studies have assessed IL-33 levels in trauma patients. Xu et al. (2017) analysed plasma sampled upon admission to the trauma ICU and at two additional time points in the first 24 h, followed by daily samples obtained until day 7 or upon discharge from the ICU. They reported that IL-33 levels were increased at admission, were highest in the first four hours after admission, and that levels were elevated for several days. Halát et al. (Halát et al. 2019) also reported elevated plasma levels of IL-33 in samples taken at admission. However, detailed early kinetic data on human IL-33 release has not yet been reported.

In order to study the dynamics of IL-33 release we have analyzed a unique collection of human plasma samples obtained serially with 2 h time resolution after a broad range of traumatic injuries. We show that IL-33 is released rapidly at detectable levels upon widespread tissue injury, in agreement with its putative role as a damage-released mediator in humans. Furthermore, we report a later increase of its decoy receptor sST2 consistent with a subsequent regulatory response.

## Methods

Trauma patients admitted to Oslo University Hospital Ullevål, a Level 1 trauma center, were included prospectively from January 2011 to January 2014. The cohort was characterized in a recent publication (Ottestad et al. 2019). Patients less than 18 years old, burn injuries, and pregnant women were excluded. Reference values were obtained from 20 healthy volunteers. The Regional committee for medical and health research ethics approved the protocol (2010/2014 REK Sør-Øst D).

Blood was drawn in EDTA-coated tubes immediately after admission to the emergency department, 2, 4, 6, and 8 h thereafter, and daily in the intensive care unit (ICU) for up to ten days. Arterial samples were preferred in order to obtain blood that was not draining from any particular injured body part. All sampling times were converted to hours post injury. The EDTA tubes were put directly in ice slush and within 15 min centrifuged at 2500 *g* for 15 min at 4 °C. Aliquots of the supernatant were immediately transferred to long-term storage in 1.8 mL sterile polypropylene tubes (NUNC CryoTubes; Catalog No. 479-6843; VWR International AS, Oslo, Norway) at -80 °C. Blood sampling and processing was handled by seven individuals dedicated to this project only.

Plasma IL-33 was measured with Bio-Plex Pro Human Th17 Cytokine IL-33 immunoassay (Bio-Rad Laboratories, Hercules, CA). sST2 was analyzed by ELISA in duplicates with Human ST2/IL-1R4 Quantikine kit (R&D Biosystems, Minneapolis, MN).

Statistical analyses were performed using GraphPad Prism version 6.0 (GraphPad Software, La Jolla, CA) and JMP 11.2.1 (SAS Institute Inc, Cary, NC). Data from NISS > 24 patients stratified according to detectable IL-33 (IL-33pos) or undetectable (IL-33neg) were compared by the Kruskal–Wallis test with Dunn’s multiple comparison test or by *χ*^2^/Fisher’s exact test as appropriate. Two-sided *p*-values < 0.05 were considered statistically significant.

Further details are given in Additional file 1: Methods.

## Results and discussion

A total of 145 trauma patients were enrolled. Two patients withdrew from the study, three were excluded due to enrolment in an interventional study, one was excluded due to age less than 18 years, and three were taken out of the analysis due to unknown time of injury (Ottestad et al. 2019). Characteristics of the 136 patients constituting the study population are given in Table 1. The majority of patients were male (74%), median age was 40 years (quartiles 27–54, range 18–94), and blunt trauma was the predominant mechanism of injury (87%). Total 30-day mortality rate was 15%. The initial blood samples were drawn at a median time of 0:10 h (quartiles 0:06–0:16) after arrival in the emergency room, corresponding to a median time of 1:15 h (0:47–1:50) after injury. The total number of samples analyzed for IL-33 was 1094, and the median number of samples per patient was 6 (quartiles 5–10, range 1–35). Arterial samples were obtained in 969 instances (89%), central venous samples in 17 (2%), and peripheral venous samples in 105 (10%). Sample type was undocumented for 3 samples (0.3%). Peripheral venous samples were obtained from a control population consisting of healthy individuals (11 male, 9 female, median age 39 years, quartiles 32–49, range 22–58). There were no significant gender or age differences between patients and healthy controls, but the wide age range seen in the patient cohort was not reflected in the control population.

**Table 1:** Clinical characteristics of trauma patients stratified according to trauma severity and detectable IL-33

		NISS < 9	NISS 9–24	NISS > 24		*p*-value*
				IL-33neg	IL-33pos	
	n	17	44	51	24	IL-33neg *vs* pos
Age	Years	35 (26–46)	36 (28–49)	44 (29–62)	39.5 (22–61)	0.2609
Male gender	n (%)	12 (71)	34 (77)	38 (75)	17 (71)	0.6024
Time after injury of first sample	Hours	0.9 (0.8–1.2)	1.1 (0.6–1.4)	1.7 (1.1–2.5)	1.2 (0.8–1.6)	***0.0119***
Penetrating trauma	n (%)	2 (12)	8 (18)	8 (16)	0 (0)	***0.049***
Major head trauma^†^	n (%)	0 (0)	7 (16)	36 (71)	16 (67)	0.7913
Isolated major head trauma^‡^	n (%)	0 (0)	5 (11)	12 (24)	0 (0)	***0.0072***
NISS	Score	2 (1–5)	14 (10–19)	41 (34–57)	49 (41–59)	0.3390
ISS	Score	2 (1–5)	10 (9–14)	29 (21–38)	42 (29–50)	***0.0278***
Number of ISS regions affected	Number	1 (1–2)	2 (2–3)	3 (2–4)	5 (4–5)	***0.0001***
Injury to ISS region:						
Head or neck	n (%)	7 (41)	12 (27)	36 (71)	18 (75)	0.7873
Face	n (%)	1 (6)	10 (23)	22 (43)	6 (25)	0.2004
Chest	n (%)	0 (0)	19 (42)	28 (55)	21 (88)	***0.0084***
Abdominal or pelvic contents	n (%)	0 (0)	7 (15)	10 (20)	21 (88)	*** < 0.0001***
Extremities or pelvic girdle	n (%)	3 (18)	19 (43)	26 (51)	21 (88)	***0.0023***
External	n (%)	12 (71)	34 (77)	43 (84)	23 (96)	0.2566
aPTT^§^	Sec	32 (31–35)	30 (29–33)	32 (29–36)	43 (38–75)	*** < 0.0001***
aPTT > 35^§^	n (%)	4 (24)	8 (19)	14 (28)	19 (83)	*** < 0.0001***
INR^ll^	Ratio	1 (1.0–1.1)	1.1 (1.0–1.1)	1.1 (1.0–1.1)	1.2 (1.1–1.4)	*** < 0.0001***
INR > 1.2^ll^	n (%)	0 (0)	3 (7)	2 (4.1)	9 (39.1)	***0.0003***
Coagulopathic (INR > 1.5 and/or aPTT > 60)	n (%)	0 (0)	1 (2.3)	2 (4.1)	7 (30.4)	***0.0037***
Transfusion of PRBC	Units	0 (0–0)	0 (0–0)	0 (0–0)	1.5 (0–10)	***0.0004***
Base excess**	mmol/L	− 2 (0–− 3.1)	− 1.9 (− 0.9–− 4.2)	− 3.4 (− 1.6–− 6.3)	− 6.2 (− 3.7–− 13.7)	***0.0306***
CK maximum first 48 h	U/L	201 (128–574)	435 (217–1356)	1254 (284–3606)	1933 (483–5398)	0.2286
LDH maximum first 48 h	U/L	242 (202–281)	369 (198–543)	320 (273–415)	599 (416–960)	***0.0009***

All data presented as median (with quartiles) or number of patients (%)

*Analysed by Kruskal–Wallis test with Dunn’s multiple comparison test or by *χ*^2^/Fisher’s exact test as appropriate with *p* values presented in bold italics if *p* < 0.05

^†^Defined as AIS score ≥ 3 in ISS region Head or neck

^‡^Defined as major head trauma with no registered injuries to ISS regions Chest, Abdominal or pelvic contents, or Extremities or pelvic girdle

^§^3 patients excluded from analysis due to known anticoagulant treatment prior to injury and 3 patients excluded due to no aPTT test performed at admission

^ll^3 patients excluded from analysis due to known anticoagulant treatment prior to injury and 1 patient excluded due to no INR test performed at admission

**9 patients excluded from analysis due to no test performed at admission

*NISS* New Injury Severity Score; *ISS* Injury Severity Score; *aPTT* activated partial thromboplastin time; *INR* International Normalized Ratio; *PRBC* packed red blood cells; *CK* creatine kinase; *LDH* lactate dehydrogenase

**Fig. 1:**
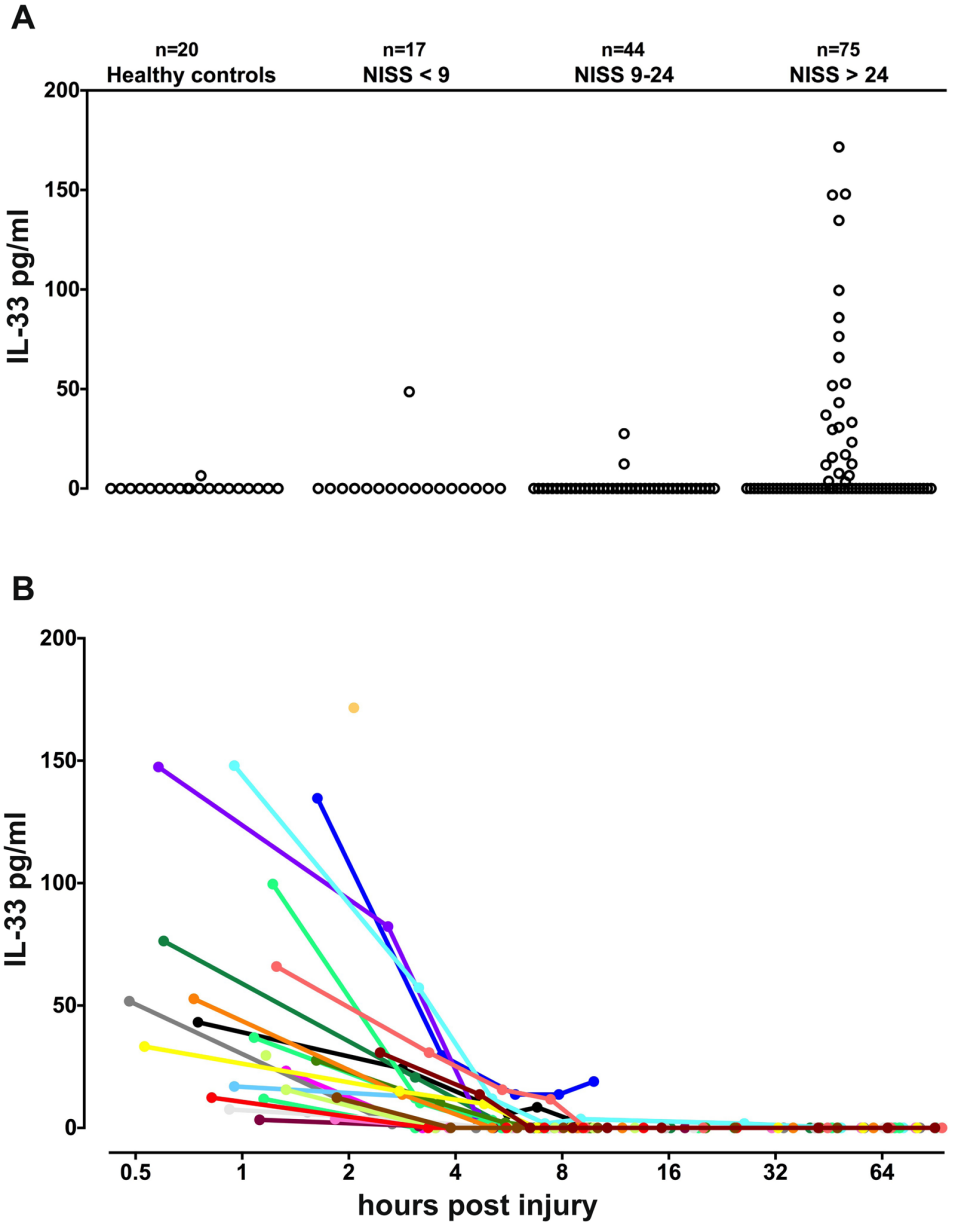
IL-33 concentrations in healthy controls and in trauma patients stratified according to New Injury Severity Score (NISS). **a** IL-33 levels at admission. **b** IL-33 concentration kinetics in individual critically injured patients (NISS > 24) with detectable IL-33 at admission (one separately colored curve for each patient)

Serial samples taken from admission at regular intervals up to ten days were analyzed. IL-33 levels above the detection limit of our assay were only seen in a small subset of patients at admission (n = 27, median 33 pg/mL, quartiles 12–76, range 3.3–172). Almost all of them were critically injured with a New Injury Severity Score (NISS) > 24 (Fig. 1a, Table 1). IL-33 was below detection limit in all but one healthy control (Fig. 1a). This is in accordance with a previous study where only a small proportion of controls had detectable IL-33 (Rivière et al. 2016). In most patients with detectable IL-33 upon admission there was a subsequent rapid decay to below detection limit as soon as 8 h after injury (Fig. 1b). IL-33 levels were always highest in the first sample after admission, and as all initial samples from these patients were obtained 0:29–2:27 h after injury, this indicates release of a pre-stored alarmin rather than de novo synthesis. Supporting this assumption, the IL-33 decay rate from the first to the second sample was proportional to the initial concentration (data not shown), *i.e.* IL-33 obeyed first order kinetics. When including all IL-33 measurements within the first 10 h after injury the calculated half-life (t_1/2_) was 1.4 h (95% CI 1.2–1.6; 64 samples from 28 patients). IL-33 was never detected beyond 12 h post injury, despite several patients suffering from post-trauma complications.

Our data appear to contradict Xu et al. (2017) who reported persistent IL-33 elevation for several days after severe trauma as well as detectable levels in healthy volunteers. The distributions of age, injury severity and injury mechanism are comparable in the two studies. We thus speculate that the observed differences in IL-33 levels may be due to different sensitivity/specificity in the detection assays, or to differences in pre-analytical conditions. To examine whether a large fraction of IL-33 was undetectable by our immunoassay, we performed supplementary experiments using immunoprecipitation and western blotting. We were not able to detect IL-33 in normal plasma or in patients with high IL-33 plasma concentration as determined by our ELISA, even when reducing the background noise by cleaning out a large fraction of albumin and immunoglobulin from samples (Additional file 1: Figure S1). It is worth noting that previous studies comparing various IL-33 immunoassays also showed low or undetectable levels of IL-33 in serum when using assays validated for use with human sera (Ketelaar et al. 2016; Rivière et al. 2016).

**Fig. 2:**
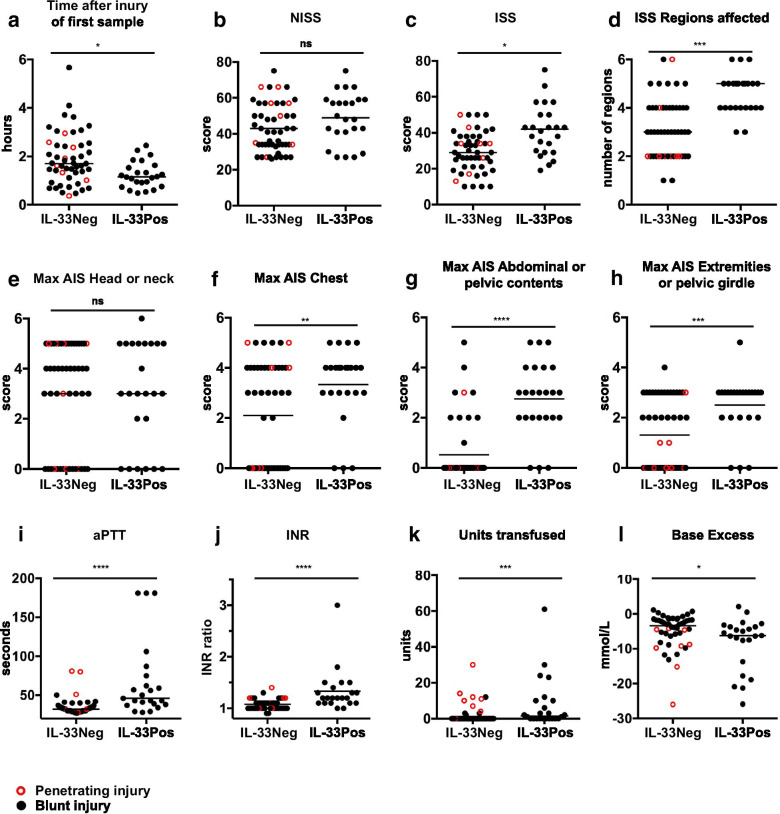
Critically injured patients (NISS > 24) stratified according to absence (IL-33neg) or presence (IL-33pos) of detectable IL-33 at admission. Differences in **a** time after injury of first sample, **b** New Injury Severity Score (NISS), **c** Injury Severity Score (ISS), **d** number of ISS regions with injuries, **e**–**h** maximum Abbreviated injury scale (AIS) severity score (with 0 representing no injury) in ISS regions Head or neck, Chest, Abdominal or pelvic contents, and Extremities or pelvic girdle, **i** activated partial thromboplastin time (aPTT) at admission, **j** international normalized ratio (INR) at admission, **k** units of packed red blood cells transfused prior to ICU admission, and **l** base excess at admission, are plotted and compared between the two groups. Black filled circles represent patients with blunt injuries, and red outlined circles represent patients with penetrating injuries. Statistical comparisons were performed with Kruskal–Wallis test with Dunn’s multiple comparison test on the patient groups, with both blunt and penetrating injuries included. Lines indicate median values. ns *p* ≥ 0.05, *p < 0.05, **p < 0.01, ***p < 0.001, ****p < 0.0001

The rapid decay rate of IL-33 made it unfeasible to correlate absolute levels to clinical characteristics, in particular because the time after injury on admission varied with several hours between patients (Table 1). Instead, we assessed whether the group of critically injured trauma patients (NISS > 24, n = 75) who had detectable IL-33 at admission (referred to as ‘IL-33pos’, n = 24) differed in terms of injuries and physiological findings from critically injured patients without detectable IL-33 (‘IL-33neg’, n = 51) within the same injury severity group. There was a significant difference in time from injury to the first sample (*p* = 0.01), with more patients in the IL-33neg group being admitted at later times after injury, suggesting missed detection due to rapid IL-33 concentration decay in some patients (Fig. 2a, Table 1). None of the 8 patients in the critical injury group who had suffered penetrating trauma had detectable IL-33 levels, however there was no significant difference regarding blunt vs. penetrating injury mechanism.

We looked closer at patient data for severity and anatomical localization of injuries. There was no significant difference (*p* = 0.34) when comparing NISS between the groups (Table 1, Fig. 2b). However, NISS differs from the original Injury Severity Score (ISS) in that the 3 most severe injuries are included *regardless* of body region. Thus, NISS correlates better with mortality than ISS (Lavoie et al. 2004), but does not differentiate between severe localized trauma and tissue damage across several body regions, the latter presumably causing more extensive release of IL-33. In accordance with this assumption, ISS was significantly higher in the IL-33pos group compared to the IL-33neg group (Fig. 2c), and the number of injured ISS body regions was substantially higher in IL-33pos patients (median 5 vs. 3, Fig. 2d) with all IL-33pos patients having injuries to 3 or more ISS regions.

We also examined effects of different anatomical injury patterns (Table 1). There were no significant differences between the IL-33pos and IL-33neg groups regarding whether they were injured or not in the ISS regions ‘Head or neck’, ‘Face’, or ‘External’, whereas a difference was seen in ‘Abdominal or pelvic contents’, ‘Chest’, and ‘Extremities or pelvic girdle’. A similar pattern was observed when we compared maximum AIS injury severity between IL-33pos and IL-33neg for each region. Using “0” as severity code for no injury, we found no significant difference in the regions ‘Head or neck’, ‘Face’, and ‘External’, whereas there were differences in ‘Abdominal or pelvic contents’ (median injury severity 3 for IL-33pos patients, 0 for IL-33neg, *p* < 0.0001), ‘Chest’ (median injury severity 4 and 2, *p* = 0.04), and ‘Extremities or pelvic girdle’ (median injury severity 3 and 1, *p* = 0.007). Finally, we explored the relative contribution of injuries in the ISS body regions in a multivariable logistic regression model for prediction of IL-33pos with maximal AIS severity code in each region as independent variables. “Informative Missing” was used to construct a coding system that allowed estimation of a predictive model despite the presence of missing values representing no injury in a particular body region. The minimum Bayesian Information Criterion was used to choose the best model through stepwise regression with backward selection. The only effect in this model was whether or not there was an injury in the region ‘Abdominal or pelvic contents’ (OR 22.3 [95% CI 5.7–117]; *p* < 0.0001; area under ROC curve = 0.82).

Previous work by us and others has shown that nuclear IL-33 is constitutively expressed in endothelial cells along the vascular tree in humans (Küchler et al. 2008; Moussion et al. 2008). Taken together with the observed kinetics in this study which point to release of pre-stored IL-33, it is likely that stressed or damaged vascular endothelium is the main source of plasma IL-33 after trauma. Acute traumatic coagulopathy and associated transfusion requirements, although multifactorial in origin, are related to the severity of endothelial injury (Dobson et al. 2015). We therefore compared admission activated partial thromboplastin time (aPTT) and prothrombin time (PT, represented by international normalized ratio, INR) in IL-33pos and IL-33neg patients, after exclusion of 3 patients with known anticoagulant treatment regimens prior to injury. A large proportion of patients had abnormal coagulation, defined as INR > 1.2 and/or aPTT > 35 s (Table 1). Importantly, both aPTT and INR were significantly higher in the IL-33pos group (Fig. 2i, j). Clinically significant coagulopathy, by some defined as INR > 1.5 and/or aPTT > 60 (Brohi et al. 2003), was only seen in 9 patients with critical injuries. Strikingly, 7 of these had detectable IL-33 at admission, including all 4 patients with an initial IL-33 level above 100 pg/mL.

Although a relatively small proportion of patients received transfusions, the IL-33pos group received significantly more units of packed red blood cells than the IL-33neg group (Fig. 2k, Table 1). Furthermore, most of the patients with high transfusion requirements in the IL-33neg group had suffered penetrating injuries (Fig. 2k). In fact, all but one of the patients with penetrating injuries received transfusions, consistent with greater degree of disruption of large vessels. Accordingly, the difference in transfusion requirement between IL-33pos and IL-33neg groups was even more pronounced when comparing only blunt injuries.

We consider it likely that the source of systemically released IL-33 is the vascular endothelium, which in humans shows strong constitutive expression of IL-33. Endothelial cells as primary source would be in agreement with the low IL-33 levels detected, as these cells represent a small tissue volume. A recent study showed that endotheliopathy can be detected as early as 5–10 min after trauma (Naumann et al. 2018), and this would fit with a process where trauma-induced endothelial damage causes an early release of alarmins. However, IL-33 is also expressed in various stromal and epithelial cells (Moussion et al. 2008; Pichery et al. 2012; Sanada et al. 2007), and contribution from other cell types is therefore also possible. In a study of patients undergoing liver resection, surgery resulted in a fivefold increase in post-operative serum IL-33 compared with pre-operative levels (Yazdani et al. 2017). Our finding that injuries in the ISS region ‘Abdominal or pelvic contents’ were particularly important for prediction of IL-33pos patients is well compatible with their results.

Although the number of deaths in our study was only 20, there were significant associations between IL-33 and survival. 16 patients died during the first 48 h after trauma, five from massive haemorrhage and nine from major head injury defined as maximum AIS ≥ 3 in ISS region Head or neck; a total of 16 died from or with major head injury. There were 46% non-survivors in the IL-33pos group and 16% in the IL-33neg group (*p* = 0.009), and admission IL-33 concentrations were significantly higher in non-survivors compared to survivors (median 7.6 vs. 0, *p* = 0.007) in those groups. IL-33 kinetics for survivors and non-survivors with and without major head injury is shown in Additional file 1: Figure S2.

**Fig. 3:**
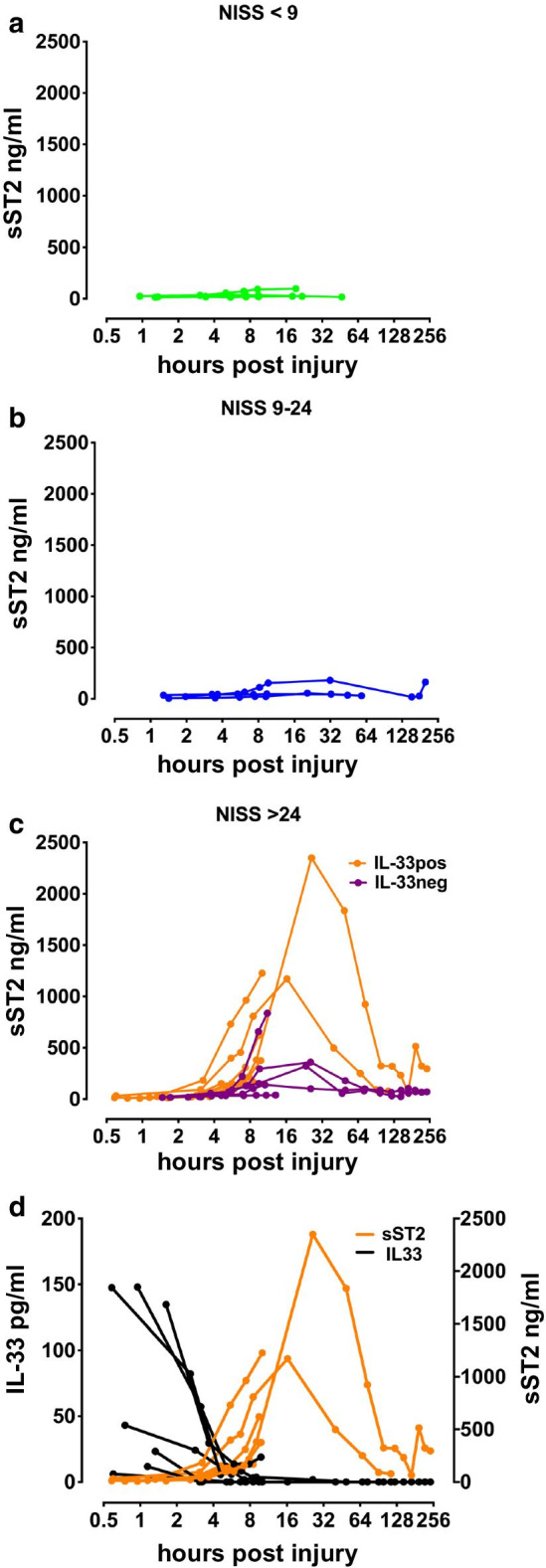
sST2 measured by ELISA in plasma in a selection of trauma patients from the cohort. Concentration curves are plotted in separate panels **a** (n = 3), **b** (n = 3), and **c** (n = 12) according to New Injury Severity Score (NISS) groups. In **c** (critical injury, NISS > 24), IL-33pos (n = 7) and IL-33neg (n = 5) patients are shown with orange and purple graphs, respectively. Panel **d** shows both IL-33 (black lines) and sST2 (orange lines) measurements in the IL-33pos NISS > 24 group in the same graph

Finally we compared early IL-33 kinetics to that of its decoy receptor sST2. As upregulation of sST2 after trauma has previously been described in several publications and the number of collected samples in the current study was large, we chose to analyze the full time course only in a few patients from each injury group and from a few healthy controls. The analyses confirmed that sST2 is abundantly expressed in normal plasma (range: 12–26 ng/ml), yet in our trauma patients we observed striking increases starting from around 2 h after injury. The amplitudes of the responses were associated with injury severity (Fig. 3a–c). In the NISS > 24 group peaks up to microgram levels were measured between 8 and 24 h after injury with subsequent decreases over the following days, with higher peak concentrations seen in patients who also had detectable IL-33 at admission (Fig. 3c). Importantly, the concentration was always lowest at the first time point, thus showing an inverse relationship to the profile of IL-33 (Fig. 3d). Moreover, the concentrations in admission samples were remarkably similar between different groups and also similar to healthy controls (Additional file 1: Figure S3). Together these observations point to an increase in protein produced de novo and secreted as a compensatory response to damage, consistent with previous data on sST2 as an inducible responder (Pascual-Figal and Januzzi 2015) upon e.g. NF-kB activation (Mildner et al. 2010).

## Conclusion

We report high-resolution kinetic data addressing systemic release of IL-33 and document a rapid surge of IL-33 after severe tissue injuries. The findings strengthen the hypothesis that IL-33 is an early mediator of the immune response after tissue injury in humans. However, the highly pleiotropic nature of this cytokine with both pro-inflammatory effects on innate immune cells and anti-inflammatory effects on T regulatory cells makes it difficult to predict net effects of the IL-33 surge. Further work in relevant experimental models is therefore needed in order to decipher the exact function of this alarmin in human trauma-induced immune responses.

## Supplementary Information

**Additional file 1.** Additional methods and figures.

## Data Availability

The data that support the findings of this study are available from Oslo University Hospital, but restrictions apply to their availability. They were used under license for the current study, and so are not publicly available. Data are however available from the authors upon reasonable request and with permission from Oslo University Hospital.

## Abbreviations

IL-33: Interleukin-33
HMGB1: High mobility group box 1 protein
NISS: New injury severity score
ISS: Injury severity score
AIS: Abbreviated injury scale
INR: International normalized ratio
PT: Prothrombin time
aPTT: Activated partial thromboplastin time
PRBC: Packed red blood cells
NF-kB: Nuclear factor kappa-light-chain-enhancer of activated B cells
