# Supplementary material for: Rapid systemic surge of IL-33 after severe human trauma: a prospective observational study
Source: Mol Med. 2021 Mar 26;27:29. doi: 10.1186/s10020-021-00288-1 (PMC8004436; doi:10.1186/s10020-021-00288-1)
Supplement: Supplementary file 1 — Additional file 1. Additional methods and figures. [file 10020_2021_288_MOESM1_ESM.pdf]

Supplementary methods

Study design

This was a prospective study of temporal changes in IL-33 level in trauma patients. It relied on convenience recruitment of trauma patients admitted to Oslo University Hospital Ullevål (OUH-U), a Level I trauma centre for 2.7 million inhabitants, i.e., half of the Norwegian population. All adult patients who met criteria for trauma team activation were eligible for enrolment. Patients less than 18 years old, burn injuries, and pregnant women were excluded. Patients were followed during 10 days, until discharge from the intensive care unit (ICU), or death, whichever event came first. Reference IL-33 values were obtained from 20 healthy volunteers.

Sample Collection

Enrolment of trauma patients was achieved from January 2011 to January 2014. Blood was drawn in 4 ml EDTA tubes as soon as possible after admission to the emergency department. Sampling through an arterial cannula was preferred to obtain blood that was not draining from any particular injured body part. Samples were also obtained as close as possible to 2, 4, 6, and 8 hours after the first sample (high time resolution samples), and then every morning in the ICU for up to ten days (low time resolution samples). The first 12 patients constituted a pilot, and in this group daily ICU samples were continued until discharge from ICU even if this occurred more than 10 days after admission. Additional arterial blood samples were drawn from five of the included trauma patients during helicopter transfer to OUH-U. These samples were stored in an insulated bag with ice packs until arrival in the emergency department.

Clinical data

Clinical data including demographics, mechanism of injury, injury severity, time of arrival in the emergency department, time of injury, physiological data at admission, and routine blood analyses were collected from the Oslo University Hospital Trauma Registry (OUH-TR). Anatomic injury was coded according to the Abbreviated Injury Scale 1990 Revision Update 1998, while all other data points were coded according to The Utstein template for uniform reporting of data following major trauma(1). Time of injury was obtained from time reported by witnesses and/or time of first call to the emergency medical communication centre. New Injury Severity Score (NISS)(2) was chosen as a measure for injury severity. NISS is a

modification of the injury severity score (ISS), an overall anatomical injury scoring system according to which each injury is assigned an abbreviated injury scale (AIS) score and allocated to one of six body regions. The 3 most severely injured body regions have their most severe AIS score squared and added together to produce the ISS. By contrast, NISS is defined as the sum of squares of the AIS scores of each of a patient's three most severe injuries regardless of body region. NISS is a better predictor of post-injury multiple organ failure and mortality than ISS but a less accurate measure of total tissue injury volume(3).

Base excess (BE) is a useful surrogate marker of hypoperfusion, and several studies have shown an exponential increase in mortality in patients with an admission BE< -6. In our cohort measurement of BE was missing in 9 patients.

IL-33 Bio-plex analyses

Analyses for IL-33 in 1094 patient plasma samples and 20 control samples were performed with a bead-based immunoassay using the Bio-Plex Pro Human Th17 Cytokine IL-33 set (Bio-Rad Laboratories, Hercules, CA) and analyzed with a Bio-Plex MAGPIX multiplex reader according to the manufacturer's instructions. All plasma samples were diluted 1:4 before analysis. Lower detection limit (LDL) in this immunoassay ranged from 1.7-4.4 pg/ml within different runs, and samples that were below LDL were assigned 0 pg/ml.

IL-33 Immunoprecipitation

4 μg of an anti-IL-33 rabbit antibody (raised against the first 15 amino acids of the N-terminal) were bound to 50 μl sheep anti-rabbit Dynabeads (Life Technologies, Oslo, Norway) by end-to-end rotation for 40 min at 4 °C. 400 μl serum from trauma patients (diluted 1:2 in PBS) or 20 μg human umbilical vein endothelial cells (HUVEC) lysate (diluted in 800 μl PBS) were incubated with the antibody-beads for 16 h at 4 °C. Immunoprecipitated IL-33 was washed 2x1 mL PBS and 2x1 mL PBS-T (0.1%) and eluted in Laemmli loading buffer before subjected to SDS-PAGE (4-20% gradient gel, Bio-Rad laboratories, Oslo, Norway) and subsequently transferred to a nitrocellulose (NC) membrane (Bio-Rad laboratories, Oslo, Norway). NC membrane was blocked in 5% non-fat dry milk in TBS-T (0.05%) and primed with Nessy-1 (Enzo Life Sciences cat. no. ALX-804-840, diluted 1:1000 in 1% milk) and incubated at 4 °C over night. Membrane was washed 4x 5 min in TBS-T. Anti-mouse IgG-HRP coupled antibody (Jackson ImmunoResearch, 1:20000 in 1% milk) was used as secondary antibody and incubated at room temperature for 2 hours.

Membrane was washed as previously described before addition of ECL western blotting substrate (Life Technologies, Oslo, Norway)

ST2 ELISA

Analyses for ST2 were performed using the Human ST2/IL-1 R4 Quantikine ELISA kit (R&D Biosystems, Minneapolis, MN). The antibodies used in this ELISA detects both membrane-bound ST2 (ST2L) and soluble ST2 (sST2), but in cell-free plasma/serum samples it is considered an accurate measure of sST2. A selection of samples from the cohort was chosen (18 patients and 6 controls) in order to include survivors/non-survivors, IL-33neg/IL-33pos and patients with different injury severity. Due to large increases in sST2 levels after trauma that extended beyond the dynamic range of detection of the ELISA, all samples were run in duplicates in two different dilutions (1:40 and 1:1000). Concentration values from samples with low levels of ST2 (<50 ng/ml) were obtained from the low dilution, and similarly values from samples with high levels (>50 ng/ml) were obtained from the highly diluted replicates. A perfect dilution linearity performance of the ELISA as described by the producer was not observed in our hands. This incongruence therefore represents some inaccuracy in absolute concentrations when using values obtained from two different dilutions but it had no impact on the overall shape of the time curves shown in Figure 3.

Routine laboratory tests

Standard laboratory tests, including aPTT, INR and base excess, were performed routinely at admission, and at later times points either as part of routine measurements in the ICU or at clinicians' discretion. Neither CK nor LDH were routinely obtained on admission, but maximum levels within first 48 hours after injury are shown in table 1. All analyses were performed according to standard operating procedures at the department of medical biochemistry, Oslo University Hospital. As part of this research project we have access to electronic records of all the laboratory analyses.

1. Ringdal KG et al. The Utstein template for uniform reporting of data following major trauma: a joint revision by SCANTEM, TARN, DGU-TR and RITG. *Scand J Trauma Resusc Emerg Med* 2008;16(1):7.

2. Osler T, Baker SP, Long W. A modification of the injury severity score that both improves accuracy and simplifies scoring. *J Trauma* 1997;43(6):922–5– discussion 925–6.

3. Balogh Z, Offner PJ, Moore EE, Biffl WL. NISS predicts postinjury multiple organ failure better than the ISS. *J Trauma* 2000;48(4):624–7– discussion 627–8.

Supplemental Figure 1

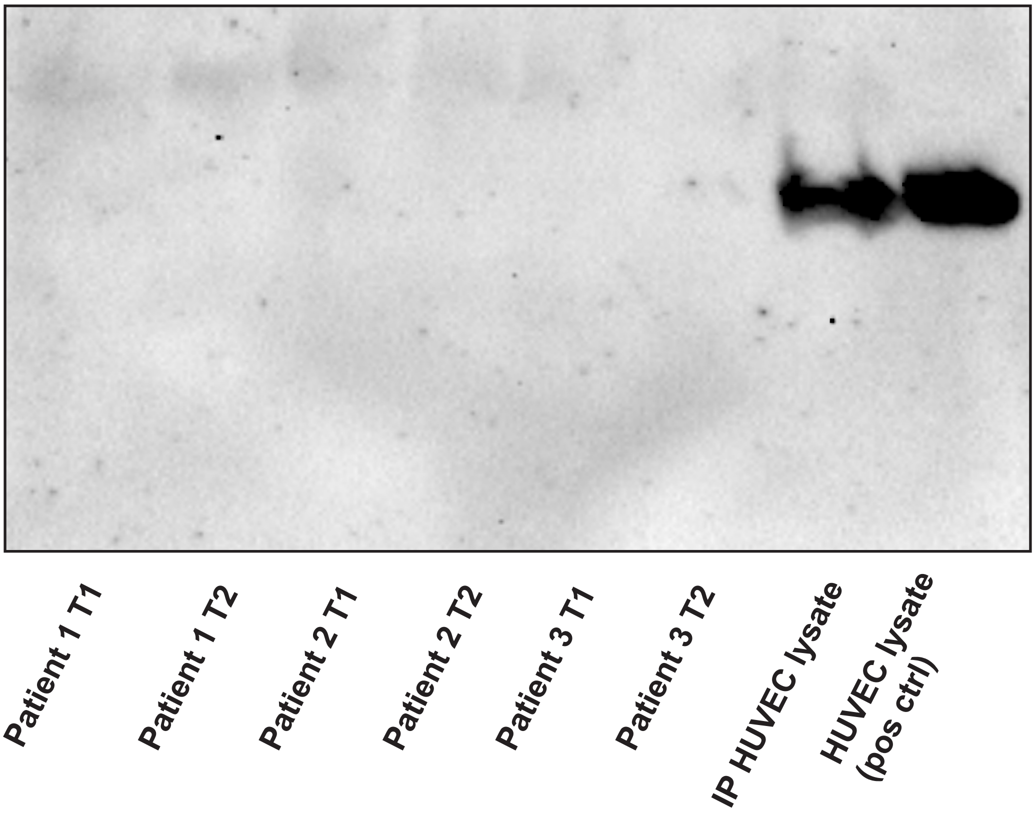

Supplemental figure 1
Immunoprecipitation of samples from 3 patients with detectable IL-33 at admission as determined by bead-based immunoassay. Two samples from each patient were analyzed; an admission sample (T1) with high IL-33 level and one subsequent sample where the IL-33 level as determined by immunoassay was low or undetectable (T2). Immunoprecipitation of lysates of human umbilical vein endothelial cells (HUVECs) and direct detection of IL-33 in HUVEC lysate were included as controls.

Supplemental Figure 2

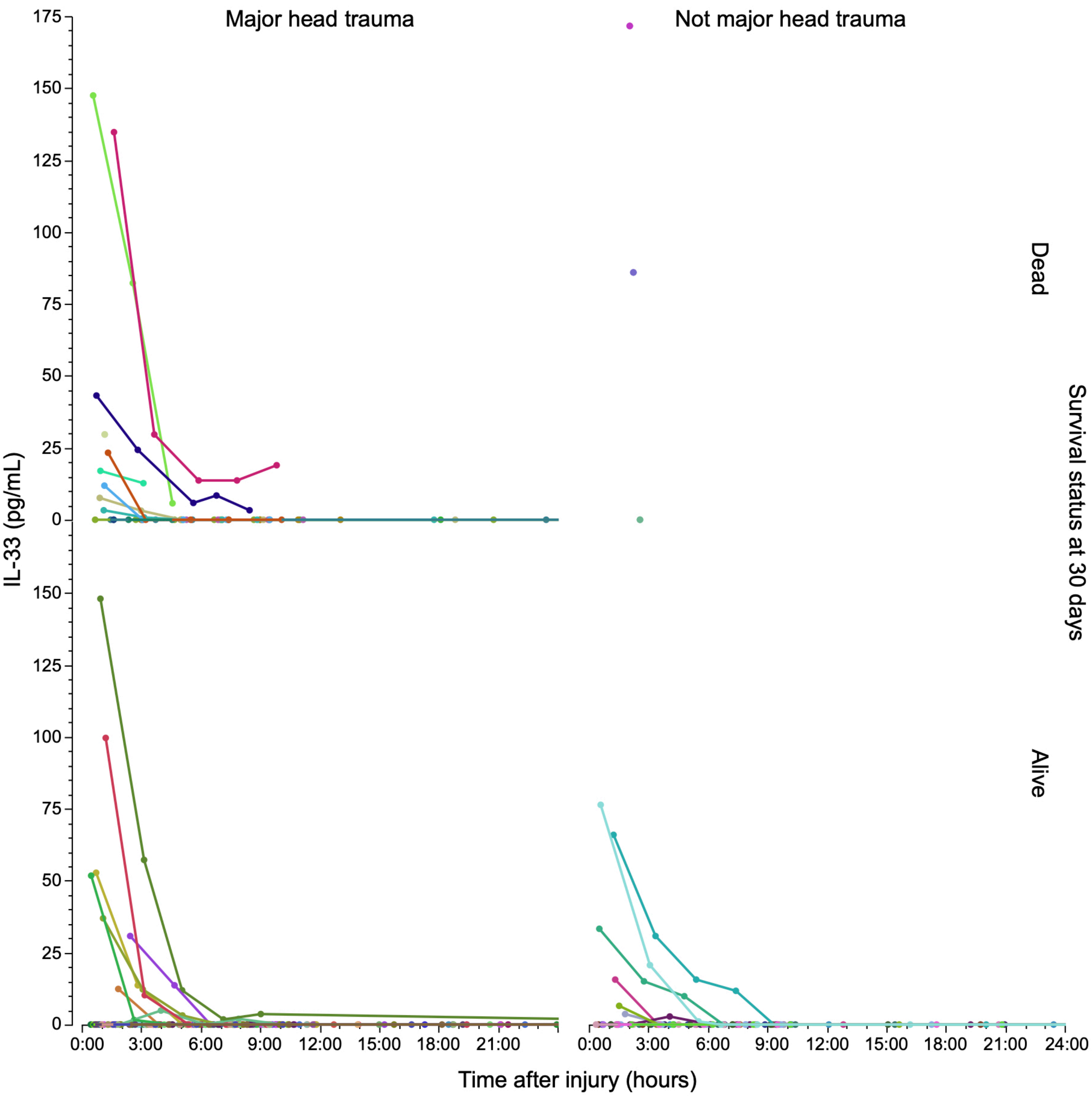

Supplemental figure 2
IL-33 concentration kinetics in individual critically injured patients (NISS>24) for survivors and non-survivors with or without major head injury (defined as maximum AIS ≥3 in ISS region Head or neck)

Supplemental Figure 3

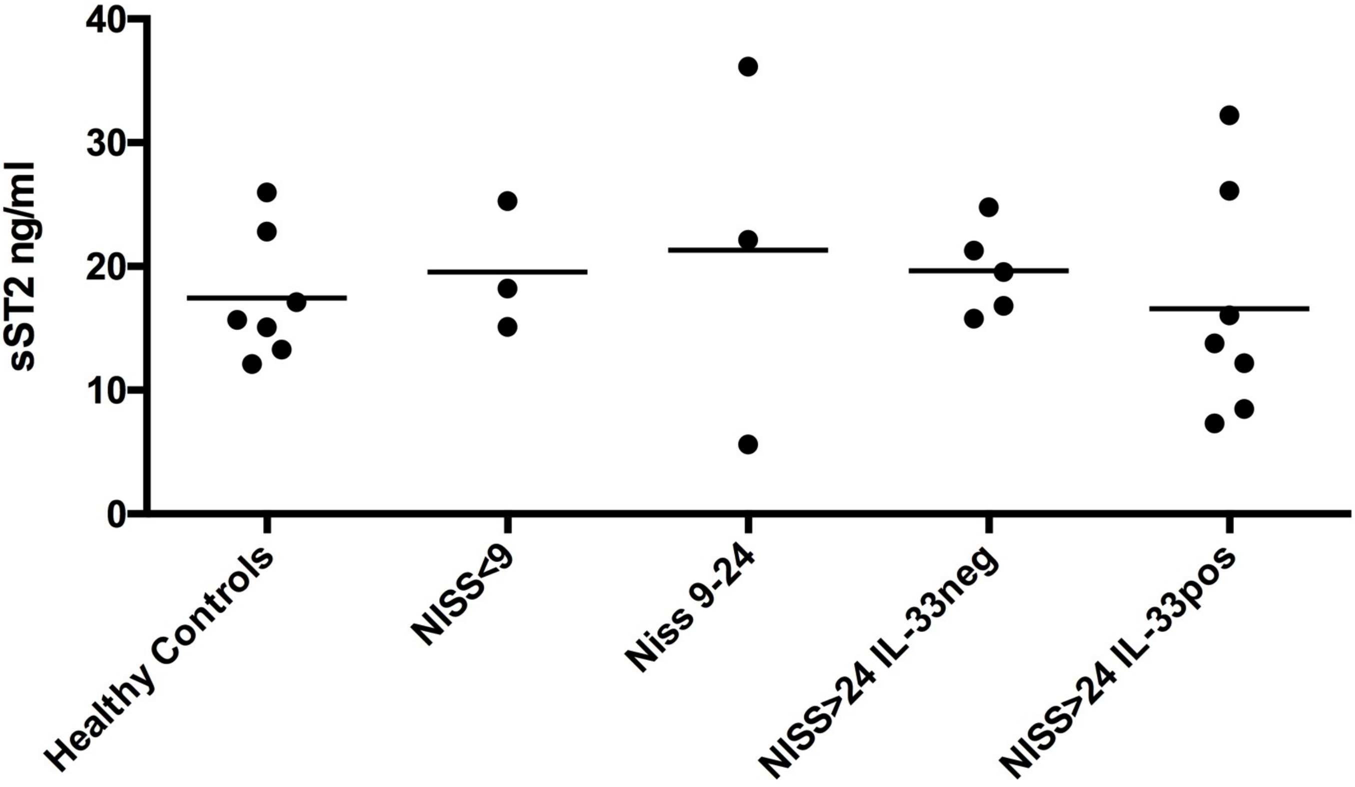

Supplemental figure 3
Plasma levels of soluble ST2 (sST2) as measured by ELISA in healthy controls and in trauma patients at admission to hospital. The trauma patients are stratified according to anatomic injury severity (New Injury Severity Scale, NISS), and to IL-33pos/IL-33neg at admission.
